# Supplementary material for: Differential Genetic and Epigenetic Effects of the KLF14 Gene on Body Shape Indices and Metabolic Traits
Source: Int J Mol Sci. 2022 Apr 9;23(8):4165. doi: 10.3390/ijms23084165 (PMC9032945; doi:10.3390/ijms23084165)

Supplementary materials

Supplementary Method

(1) Definitions of hypertension, diabetes mellitus, obesity, current smoking, and metabolic syndrome

(2) Measurement of body fat percentage (BFP)

(3) Formulas for body-shape indices: a body-shape index (ABSI), hip index (HI) and waist hip index (WHI)

(4) Formulas for the product of triglyceride and fasting plasma glucose (the TyG index), TyG with adiposity status (TyG-body mass index [BMI] and TyG waist circumference)

Supplementary Table 1. Association between *KLF14* rs4731702 genotypes and body-shape indices and metabolic traits in Taiwan Biobank participants

Supplementary Table 2. Association between *KLF14* rs4731702 genotypes and body-shape indices and metabolic traits according to obesity status

Supplementary Table 3. Association between *KLF14* rs4731702 genotypes and body-shape indices and metabolic traits according to obesity status in male participants

Supplementary Table 4. Association between *KLF14* DNA methylation status and *KLF14* variants and body-shape indices and metabolic traits

Supplementary Table 5. Association of the cg08097417 methylation status with body-shape indices and metabolic traits according to sex

Supplementary Table 6. Association of the cg08097417 methylation status with body-shape indices and metabolic traits according to obesity status

Supplementary Table 7. Association between *KLF14* rs4731702 genotypes and cg08097417 methylation status and metabolically healthy and unhealthy phenotypes in obese Taiwan Biobank participants

Supplementary Figure 1. Regional association studies for genetic variants at positions between 130.3 to 130.5 mega-base on chromosome *7q32.2* for HDL-cholesterol levels.

Supplementary Figure 2. Regional association studies for genetic variants at positions between 130.3 to 130.5 mega-base on chromosome *7q32.2* for triglyceride levels.

Supplementary Figure 3. Regional association studies for genetic variants at positions between 130.3 to 130.5 mega-base on chromosome *7q32.2* for mean blood pressure.

Supplementary Figure 4. Regional association studies for genetic variants at positions between 130.3 to 130.5 mega-base on chromosome *7q32.2* for metabolic syndrome.

Supplementary Figure 5. Regional association studies for genetic variants at positions between 130.3 to 130.5 mega-base on chromosome *7q32.2* for body mass index.

Supplementary Figure 6. Regional association studies for genetic variants at positions between 130.3 to 130.5 mega-base on chromosome *7q32.2* for waist circumference.

Supplementary Figure 7. Regional association studies for genetic variants at positions between 130.3 to 130.5 mega-base on chromosome *7q32.2* for a body-shape index.

Supplementary Figure 8. Regional association studies for genetic variants at positions between 130.3 to 130.5 mega-base on chromosome *7q32.2* for hip circumference.

Supplementary Figure 9. Regional association studies for genetic variants at positions between 130.3 to 130.5 mega-base on chromosome *7q32.2* for hip index.

Supplementary Figure 10. Regional association studies for genetic variants at positions between 130.3 to 130.5 mega-base on chromosome *7q32.2* for body fat percentage.

Supplementary Figure 11. Linkage disequilibrium map of the *KLF14* gene upstream region for lead single-nucleotide polymorphisms of the study phenotypes

Supplementary Methods

(1) *Definitions of hypertension, diabetes mellitus, obesity, current smoking and metabolic syndrome*
Hypertension was defined as systolic blood pressure (BP) of ≥140 mmHg, diastolic BP of ≥90 mmHg or a self-reported history of hypertension. Diabetes mellitus (DM) was defined as a fasting plasma glucose level of ≥126 mg/dL, a glycohemoglobin value of ≥6.5%, or a self-reported history of DM. Obesity was defined as a BMI of ≥25 kg/m^2. Current smoking was defined as regular cigarette smoking at the time of survey.

Because medication histories were unavailable, metabolic syndrome characteristics were based on the recent update of the third report of the National Cholesterol Education Program's Adult Treatment Panel III criteria (Grundy et al., 2005) with modifications. Participants with three or more of the following attributes are typically defined as having metabolic syndrome: (1) BP of ≥130/85 mmHg or a history of hypertension; (2) triglyceride level of ≥150 mg/dL; (3) high-density lipoprotein cholesterol level of <40 mg/dL for men or <50 mg/dL for women; (4) fasting plasma glucose of ≥100 mg/dL or a history of DM; and (5) waist circumference of >90 cm for men or >80 cm for women.

Reference:
Grundy SM, Cleeman JI, Daniels SR, Donato KA, Eckel RH, Franklin BA, et al. Diagnosis and management of the metabolic syndrome: an American Heart Association/National Heart, Lung, and Blood Institute Scientific Statement. Circulation. 2005;112:2735-52.

(2) *Measurement of body fat percentage (BFP)*
BFP was measured by "Body Composition Analyzer BC-420MA" TANITA, a device that sends a weak electric current through the body to measure the impedance (electrical resistance) of the body. After switched on the analyzer, we enter the weight of the clothes (it can be entered in the range of 0.0–10.0 kg) and then the participants stand on the electrode, barefoot, with both legs parallel to the electrodes facing the weighing scale without bending the legs. Then input the ID number, select the body type, gender, age and height of the participants. The results are printed automatically.

(3) *Formulas for body shape indices: a body shape index (ABSI), hip index (HI) and waist hip index (WHI)*

The anthropometric measurements were converted to allometric body shape indices. The published formulas for ABSI and HI have previously been derived for participants in the National Health and Nutrition Examination Survey (NHANES) and the formula for WHI was previously reported. (Christakoudi et al., 2021)

$ABSI = WC * Weight^{-2/3} * Height^{5/6}$.

$HI = HC * Weight^{-0.482} * Height^{0.310}$.

$WHI = WHR * [\text{Weight (kg)}/\text{Height}^2 \text{(cm)}]^{-1/4}$.

Reference:

Christakoudi S, Evangelou E, Riboli E, Tsilidis KK. GWAS of allometric body-shape indices in UK Biobank identifies loci suggesting associations with morphogenesis, organogenesis, adrenal cell renewal and cancer. Scientific reports. 2021;11:10688.

(4) *Formulas for the product of triglyceride and fasting plasma glucose (the TyG index), TyG with adiposity status (TyG-body mass index [BMI] and TyG waist circumference)*

TyG index has been revealed to be an efficient marker for early identification of insulin resistance (Guerrero-Romero et al., 2010, Du et al., 2014). BMI and waist circumference are simple, inexpensive and noninvasive anthropometric parameters and are commonly adopted as useful indicators of obesity and other metabolic risk. A combination of obesity and TyG can potentially identify IR more strongly than other surrogate markers. Thus, we use TyG and TyG related parameters (TyG BMI and TyG WC) as surrogate markers of IR. TyG related parameters were calculated as follows: TyG index: Ln [TG (mg/dL) × FPG (mg/dL)/^{2}], TyG-BMI: TyG index × BM, TyG-WC: TyG index × WC (Er et al., 2016).

References:

Guerrero-Romero F, Simental-Mendia LE, Gonzalez-Ortiz M, Martinez-Abundis E, Ramos-Zavala MG, Hernandez-Gonzalez SO, et al. The product of triglycerides and glucose, a simple measure of insulin sensitivity. Comparison with the euglycemic hyperinsulinemic clamp. The Journal of clinical endocrinology and metabolism. 2010; 95:3347–3351.

Du T, Yuan G, Zhang M, Zhou X, Sun X, Yu X. Clinical usefulness of lipid ratios, visceral adiposity indicators, and the triglycerides and glucose index as risk markers of insulin resistance. Cardiovascular Diabetology. 2014;13:146.

Er LK, Wu S, Chou HH, Hsu LA, Teng MS, Sun YC, et al. Triglyceride Glucose-Body Mass Index Is a Simple and Clinically Useful Surrogate Marker for Insulin Resistance in Nondiabetic Individuals. PLoS One. 2016;11:e0149731.

Supplementary Table 1. Association between *KLF14* rs4731702 genotypes and body-shape indices and metabolic traits in Taiwan Biobank participants

	beta	SE	*P* value
Age (years)	0.0186	0.0580	0.7479
Body-shape indices			
Body height (cm)	-0.0612	0.0298	0.0402
Body weight (kg)	-0.0438	0.0244	0.0732
Hip circumference (cm)	0.1014	0.0205	7.43×10^{-7}
Waist circumference (cm)	0.1193	0.0276	1.5×10^{-5}
Waist hip ratio	0.0002	0.0003	0.4331
Body fat percentage (%)	0.0511	0.0137	0.0002
Body mass index (kg/m^2)	0.0898	0.0197	5.2×10^{-6}
ABSI	0.0117	0.0025	2.15×10^{-6}
WHI	0.0009	0.0013	0.4763
HI	0.0201	0.0027	1.26×10^{-13}
Blood pressure and heart rate			
Mean heart rate† (/min)	-0.0279	0.0523	0.5937
Systolic BP† (mmHg)	-0.3788	0.0833	5.00×10^{-6}
Diastolic BP† (mmHg)	-0.2894	0.0543	9.70×10^{-8}
Mean BP† (mmHg)	-0.3192	0.0597	8.75×10^{-8}
Lipid profiles			
Total cholesterol§ (mmol/L)	0.0003	0.0004	0.5122
HDL cholesterol§ (mmol/L)	0.0038	0.0005	6.69×10^{-14}
LDL cholesterol§ (mmol/L)	-0.0003	0.0006	0.6471
Triglyceride§ (mmol/L)	-0.0093	0.0012	1.18×10^{-14}
Glucose metabolism			
Fasting plasma glucose‡ (mmol/L)	-0.2390	0.0824	0.0037
HbA1c‡ (%)	-0.0106	0.0033	0.0013
Insulin resistance surrogate markers			
TyG index§,‡	-275.84	54.41	3.99×10^{-7}
TyG-BMI§,‡ ($\times 10^3$)	-6932	1428	1.00×10^{-6}
TyG-WC§,‡ ($\times 10^3$)	-21873	4940	1.00×10^{-5}
Atherosclerotic risk factors			
Diabetes mellitus (%)	-0.0706	0.0199	0.0004
Hypertension (%)	-0.0581	0.0146	7.20×10^{-5}
Metabolic syndrome (%)	-0.1089	0.0153	9.92×10^{-13}

Abbreviations, adjusted condition and subjects recruited for analysis as in Figure 1 and Table 2.
P: adjusted for age, sex, BMI, and current smoking; Age: adjusted for sex, BMI and current smoking; and BMI: adjusted for age, sex and smoking. † hypertension, ‡ diabetes mellitus, and § hyperlipidemia.
Significance was defined as a *P* value of $< 0.05/(131+27) = 3.16 \times 10^{-4}$

Supplementary Table 2. Association between *KLF14* rs4731702 genotypes and body-shape indices and metabolic traits according to obesity status

Clinical and laboratory parameters	Non - obesity (n = 49,943)			Obesity (n = 28,799)		
	beta	SE	*P* value	beta	SE	*P* value
Age (years)	0.0176	0.0727	0.8083	0.0432	0.0935	0.6439
Body-shape indices						
Body height (cm)	-0.1079	0.0372	0.0037	0.0148	0.0497	0.7661
Body weight (kg)	-0.0785	0.0268	0.0033	0.0088	0.0466	0.8497
Hip circumference (cm)	0.0530	0.0243	0.0288	0.1826	0.0368	7.15×10^{-7}*
Waist circumference (cm)	0.0160	0.0336	0.6342	0.2941	0.0474	5.62×10^{-10}**
Waist hip ratio	-0.0003	0.0004	0.4056	0.0012	0.0005	0.0125
Body fat percentage (%)	0.0468	0.0159	0.0033	0.0707	0.0223	0.0016
Body mass index (kg/m^2)	0.0151	0.0129	0.2412	0.0693	0.0249	0.0054
ABSI	0.0041	0.0032	0.2088	0.0245	0.0038	1.1×10^{-10}**
WHI	-0.0014	0.0017	0.3905	0.0050	0.0020	0.0136
HI	0.0155	0.0033	2.26×10^{-6}	0.0269	0.0046	6.36×10^{-9}
Blood pressure and heart rate						
Mean heart rate† (/min)	0.0998	0.0635	0.1161	-0.2897	0.0916	0.0016**
Systolic BP† (mmHg)	-0.3109	0.1005	0.0020	-0.5082	0.1481	6.02×10^{-4}
Diastolic BP† (mmHg)	-0.2287	0.0651	4.42×10^{-4}	-0.4123	0.0976	2.40×10^{-5}
Mean BP† (mmHg)	-0.2561	0.0717	3.57×10^{-4}	-0.4443	0.1068	3.20×10^{-5}
Lipid profiles						
Total cholesterol§ (mmol/L)	0.0005	0.0005	0.3761	-0.0001	0.0007	0.9367
HDL cholesterol§ (mmol/L)	0.0033	0.0006	3.25×10^{-7}	0.0048	0.0008	1.01×10^{-8}
LDL cholesterol§ (mmol/L)	-0.0002	0.0008	0.7853	-0.0004	0.0011	0.7096
Triglyceride§ (mmol/L)	-0.0078	0.0014	5.23×10^{-8}	-0.0119	0.0021	2.22×10^{-8}
Glucose metabolism						
Fasting plasma glucose‡ (mmol/L)	-0.1275	0.0870	0.1429	-0.4367	0.1695	0.0100
HbA1c‡ (%)	-0.0069	0.0035	0.0505	-0.0172	0.0066	0.0092
Insulin resistance surrogate markers						
TyG§, ‡	-218.40	55.05	7.30×10^{-5}	-385.54	116.95	0.0010
TyG-BMI§, ‡ (× 10^3)	-4907	1266	1.06×10^{-4}	-10842	3331	0.0011
TyG-WC§, ‡ (× 10^3)	-17,044	4663	2.57×10^{-4}	-31,323	11133	0.0049
Atherosclerotic risk factors						
Diabetes mellitus (%)	-0.0759	0.0306	0.0133	-0.0650	0.0260	0.0123
Hypertension (%)	-0.0606	0.0208	0.0036	-0.0538	0.0205	0.0086
Metabolic syndrome (%)	-0.0952	0.0250	1.41×10^{-4}	-0.1140	0.0192	2.80×10^{-9}

Abbreviations, adjusted condition and subjects recruited for analysis as in Figure 1 and Table 2.
P: adjusted for age, sex, BMI and current smoking and age: adjusted for sex, BMI and current smoking. † hypertension, ‡ diabetes mellitus, and § hyperlipidemia.
Significance was defined as a *P* value of $< 0.05/(131+27) = 3.16 \times 10^{-4}$
t-test : ** P < 0.001, * P < 0.01

Supplementary Table 3. Association between *KLF14* rs4731702 genotypes and body-shape indices and metabolic traits according to obesity in male

Clinical and laboratory parameters	Male, Non-obese (n = 14,266)			Male, Obese (n = 14,217)		
	beta	SE	*P* value*	beta	SE	*P* value*
Age (years)	0.0536	0.1453	0.7121	-0.0547	0.1366	0.6886
Body-shape indices						
Body height (cm)	-0.0769	0.0747	0.3036	0.0804	0.0735	0.2740
Body weight (kg)	-0.0636	0.0575	0.2687	0.0792	0.0707	0.2626
Hip circumference (cm)	-0.0241	0.0439	0.5834	0.0573	0.0486	0.2384
Waist circumference (cm)	-0.0659	0.0545	0.2264	0.1229	0.0572	0.0319
Waist hip ratio	-0.0005	0.0005	0.3765	0.0006	0.0005	0.2421
Body fat percentage (%)	0.0564	0.0355	0.1120	0.0325	0.0345	0.3473
Body mass index (kg/m^2)	-0.0027	0.0223	0.9051	0.0259	0.0338	0.4435
ABSI	-0.0047	0.0048	0.3265	0.0074	0.0043	0.0852
WHI	-0.0023	0.0025	0.3602	0.0026	0.0024	0.2686
HI	0.0008	0.0056	0.8839	0.0033	0.0058	0.5730
Blood pressure and heart rate						
Mean heart rate† (/min)	0.0610	0.1307	0.6409	-0.2846	0.1383	0.0396
Systolic BP† (mmHg)	-0.3446	0.1912	0.0715	-0.4900	0.2091	0.0191
Diastolic BP† (mmHg)	-0.2766	0.1266	0.0289	-0.4147	0.1437	0.0039
Mean BP† (mmHg)	-0.2993	0.1372	0.0291	-0.4398	0.1545	0.0044
Lipid profiles						
Total cholesterol§ (mmol/L)	0.0001	0.0010	0.9380	-0.0002	0.0010	0.8117
HDL cholesterol§ (mmol/L)	-0.0017	0.0012	0.1600	0.0024	0.0012	0.0410
LDL cholesterol§ (mmol/L)	0.0014	0.0015	0.3691	-0.0007	0.0015	0.6417
Triglyceride§ (mmol/L)	0.0006	0.0029	0.8313	-0.0072	0.0031	0.0211
Glucose metabolism						
Fasting plasma glucose‡ (mmol/L)	0.0240	0.1913	0.9001	-0.2691	0.2518	0.2852
HbA1c‡ (%)	0.0014	0.0080	0.8641	-0.0137	0.0097	0.1578
Insulin resistance surrogate markers						
TyG§, ‡	-106.08	135.62	0.4341	-237.72	195.68	0.2244
TyG-BMI§, ‡ (× 10^3)	-2575	3120	0.4093	-6915	5561	0.2137
TyG-WC§, ‡ (× 10^3)	-10,104	11508	0.3800	-20479	18,886	0.2782
Atherosclerotic risk factors						
Diabetes mellitus (%)	-0.0302	0.0483	0.5310	-0.0402	0.0368	0.2750
Hypertension (%)	-0.0534	0.0336	0.1124	-0.0757	0.0282	0.0073
Metabolic syndrome (%)	-0.0148	0.0470	0.7535	-0.0819	0.0276	0.0030

Abbreviations, adjusted condition and subjects recruited for analysis as in Figure 1 and Table 2.
*P**: adjusted for age, BMI, and current smoking; Age: adjusted for BMI and current smoking; and BMI: adjusted for age and smoking. † hypertension, ‡ diabetes mellitus, and § hyperlipidemia.
Significance was defined as a *P* value of < 0.05/(131+27) = 3.16 × 10^{-4}

Supplementary Table 4. Association between *KLF14* DNA methylation levels and *KLF14* variants and age

						P values						
IlmnID	Base position	UCSC_RefGene_Name	UCSC_RefGene_Group	Relation_to_UCSC_CpG_Island	β values of DNA methylation levels#	rs3996352 genotypes	rs1364422 genotypes	rs4731702 genotypes	rs972283 genotypes	Age*	Age**	Age***
cg14788130	130380731	*KLF14*			0.8857 (0.8774 - 0.8949)	0.261	0.8339	0.1262	0.5212	0.0462	0.0169	0.5952
cg15769180	130391882	*KLF14*			0.8254 (0.8068 - 0.8441)	0.5455	0.4525	0.5219	0.2336	1.29×10^{-42}	5.59×10^{-15}	1.17×10^{-30}
cg16271549	130406947	*KLF14*			0.2351 (0.2114 - 0.2604)	0.2294	0.3614	0.3184	0.5106	0.6924	0.622	0.9289
cg19027884	130414263	*KLF14*		N-Shelf	0.6517 (0.6276 - 0.6756)	0.2402	0.9017	0.3096	0.2383	3.60×10^{-8}	0.0007	1.11×10^{-5}
cg03578375	130417085	*KLF14*		N-Shore	0.8960 (0.8838 - 0.9076)	0.9435	0.2751	0.91	0.7339	0.0274	0.0458	0.2576
cg23399222	130417715	*KLF14*	1st Exon;3'UTR	N-Shore	0.8099 (0.7900 - 0.8289)	0.2847	0.9051	0.3426	0.7956	0.1301	0.2178	0.3626
cg04528819	130418315	*KLF14*	1st Exon	Island	0.1764 (0.1571 - 0.1954)	0.4777	0.8799	0.5894	0.7377	8.47×10^{-119}	5.07×10^{-63}	1.78×10^{-59}
cg20426994	130418324	*KLF14*	1st Exon	Island	0.1140 (0.0959 - 0.1341)	0.1787	0.6853	0.1912	0.1406	2.08×10^{-111}	3.33×10^{-59}	3.14×10^{-55}
cg00094518	130418549	*KLF14*	1st Exon	Island	0.1936 (0.1624 - 0.2283)	0.7445	0.406	0.7645	0.663	4.15×10^{-141}	4.19×10^{-77}	5.20×10^{-70}
cg09823095	130419042	*KLF14*	TSS200	Island	0.0197 (0.0150 - 0.0249)	0.3876	0.1777	0.4113	0.6812	2.26×10^{-17}	1.25×10^{-10}	1.44×10^{-8}
cg25109431	130419057	*KLF14*	TSS200	Island	0.0186 (0.0146 - 0.0226)	0.8381	0.4882	0.8799	0.4485	8.09×10^{-7}	0.0061	2.68×10^{-5}
cg21449170	130419062	*KLF14*	TSS200	Island	0.0567 (0.0485 - 0.0661)	0.4869	0.7431	0.4766	0.4567	3.69×10^{-30}	1.87×10^{-16}	8.18×10^{-16}
cg05651960	130419064	*KLF14*	TSS200	Island	0.0533 (0.0461 - 0.0617)	0.1841	0.0326	0.3231	0.1074	7.98×10^{-12}	4.87×10^{-8}	1.96×10^{-5}
cg18751682	130419066	*KLF14*	TSS200	Island	0.0471 (0.0397 - 0.0558)	0.7911	0.7577	0.8534	0.9897	9.20×10^{-25}	1.11×10^{-16}	4.32×10^{-10}
cg08097417	130419133	*KLF14*	TSS1500	Island	0.1741(0.1492 - 0.1998)	0.5339	0.2318	0.6571	0.416	3.83×10^{-253}	9.28×10^{-135}	2.97×10^{-127}

cg07955995	130419159	*KLF14*	TSS1500	Island	0.0573 (0.0434 - 0.0749)	0.9929	0.0835	0.967	0.6541	4.20×10^{-153}	9.22×10^{-78}	6.92×10^{-79}
cg22285878	130419173	*KLF14*	TSS1500	Island	0.0655 (0.0533 - 0.0793)	0.6426	0.197	0.7456	0.6767	8.16×10^{-59}	3.62×10^{-38}	9.58×10^{-24}
cg21520933	130419340	*KLF14*	TSS1500	Island	0.1203 (0.0903 - 0.1630)	0.4646	0.0512	0.3312	0.2751	0.0853	0.1357	0.3602
cg06533629	130419370	*KLF14*	TSS1500	Island	0.1239 (0.1039 - 0.1449)	0.0469	0.4193	0.0325	0.3854	3.77×10^{-8}	8.07×10^{-6}	0.0009
cg08719712	130419485	*KLF14*	TSS1500	S-Shore	0.1294 (0.1129 - 0.1481)	0.0745	0.1835	0.0501	0.1083	5.64×10^{-38}	7.58×10^{-18}	2.66×10^{-22}
cg02778245	130419514	*KLF14*	TSS1500	S-Shore	0.0917 (0.0790 - 0.1050)	0.7298	0.6379	0.7621	0.3155	3.21×10^{-10}	1.26×10^{-5}	5.89×10^{-6}
cg18645297	130419594	*KLF14*	TSS1500	S-Shore	0.0840 (0.0663 - 0.1049)	0.4737	0.6493	0.4825	0.2474	0.1704	0.1351	0.6828
cg17317023	130419614	*KLF14*	TSS1500	S-Shore	0.0907 (0.0766 - 0.1058)	0.1595	0.1022	0.265	0.443	1.07×10^{-16}	4.87×10^{-9}	4.61×10^{-9}
cg06038655	130419675	*KLF14*	TSS1500	S-Shore	0.2058 (0.1725 - 0.2428)	0.715	0.1339	0.6919	0.8807	3.21×10^{-7}	1.82×10^{-5}	0.0035
cg03914913	130419754	*KLF14*	TSS1500	S-Shore	0.0834 (0.0676 - 0.1024)	0.6374	0.7033	0.5772	0.5944	0.319	0.3018	0.6847
cg09529138	130419792	*KLF14*	TSS1500	S-Shore	0.1487 (0.1258 - 0.1742)	0.1558	0.3704	0.1097	0.4827	0.2065	0.4608	0.2943
cg02423044	130419932	*KLF14*	TSS1500	S-Shore	0.2096 (0.1785 - 0.2467)	0.9079	0.1863	0.9502	0.6601	0.371	0.3216	0.7843
cg02385110	130421878	*KLF14*		S-Shelf	0.7276 (0.7001 - 0.7568)	0.2608	0.372	0.2758	0.2378	0.127	0.1431	0.4641
cg24042772	130478741	*KLF14*			0.8602 (0.8468 - 0.8719)	0.4067	0.9123	0.4398	0.1147	8.09×10^{-12}	2.48×10^{-5}	4.49×10^{-8}
cg08867923	130499719	*KLF14*			0.5786 (0.5495 - 0.6069)	0.2891	0.5253	0.2776	0.6012	4.40×10^{-8}	4.49×10^{-5}	7.94×10^{-5}
cg20631720	130507724	*KLF14*			0.2264 (0.1892 - 0.2658)	0.838	0.3898	0.8495	0.5819	5.78×10^{-19}	7.88×10^{-5}	3.40×10^{-19}
cg20789819	130516373	*KLF14*			0.1511 (0.1283 - 0.1732)	0.4298	0.3613	0.4675	0.3296	1.07×10^{-104}	2.40×10^{-46}	1.10×10^{-60}

The UCSC_RefGene_Name, UCSC_RefGene_Group, Relation_to_UCSC_CpG_Island, and Regulatory_Feature_Group are derived from the UCSC (University of California Santa Cruz) database. https://genome.ucsc.edu/

* *P* value for age using total population for analysis

** *P* value for age using female participants for analysis
*** *P* value for age using female participants for analysis
# Median (interquartile range)

Supplementary Table 5. Association of the cg08097417 methylation status with body-shape indices and metabolic traits according to sex

Clinical and laboratory parameters	Male (n = 762)				Female (n = 874)			
	beta	SE	*P* value**	*P* value*	beta	SE	*P* value**	*P* value*
Age (years)	0.0154	0.0005	6.84×10^{-124}	-	0.0153	0.0006	3.15×10^{-119}	-
Body-shape indices								
Body height (cm)	-0.0083	0.0014	1.47×10^{-9}	0.7015	-0.0088	0.0014	1.04×10^{-9}	0.6823
Body weight (kg)	-0.0094	0.0016	3.96×10^{-9}	0.6703	-0.0112	0.0019	5.40×10^{-9}	0.7139
Hip circumference (cm)	-0.0107	0.0022	2.00×10^{-6}	0.1958	-0.0102	0.0021	2.00×10^{-6}	0.3230
Waist circumference (cm)	0.0064	0.0018	3.82×10^{-4}	0.678	0.0075	0.0015	3.48×10^{-7}	0.6819
Waist hip ratio	1.3487	0.1783	1.12×10^{-13}	0.1667	1.0214	0.1315	2.30×10^{-14}	0.3481
Body fat percentage (%)	0.002	0.0028	0.4861	0.0487	0.0002	0.0036	0.9569	0.0761
Body mass index (kg/m^2)	-0.0062	0.0027	0.0198	0.2756	0.0045	0.0022	0.0413	0.5934
ABSI	0.1145	0.0206	4.02×10^{-8}	0.716	0.0987	0.0152	1.26×10^{-10}	0.6763
WHI	0.2926	0.0399	5.50×10^{-13}	0.1841	0.2187	0.0292	1.56×10^{-13}	0.3986
HI	-0.0314	0.0185	0.0891	0.1123	-0.034	0.0164	0.0382	0.3566
Blood pressure and heart rate								
Mean heart rate† (/min)	-0.0008	0.0019	0.6855	0.1633	-0.0047	0.0019	0.0147	0.5480
Systolic BP† (mmHg)	0.0031	0.0006	3.15×10^{-7}	0.8515	0.0053	0.0006	1.45×10^{-20}¶¶	0.0013
Diastolic BP† (mmHg)	0.003	0.0009	6.05×10^{-4}	0.3545	0.0036	0.0009	9.10×10^{-5}	0.0886
Mean BP† (mmHg)	0.0037	0.0008	9.00×10^{-6}	0.5046	0.0055	0.0008	1.28×10^{-11}	0.0111
Lipid profiles								
Total cholesterol§ (mmol/L)	0.1489	0.1131	0.1883	0.1123	0.6792	0.1015	4.02×10^{-11}¶¶	0.7140
HDL cholesterol§ (mmol/L)	0.0492	0.0969	0.6119	0.8618	0.1364	0.0906	0.1325	0.8499
LDL cholesterol§ (mmol/L)	0.0056	0.0741	0.9394	0.2975	0.3235	0.0676	2.00×10^{-6}¶	0.4901
Triglyceride§ (mmol/L)	0.0746	0.0395	0.0589	0.2516	0.1627	0.0382	2.30×10^{-5}	0.5189
Glucose metabolism								
Fasting plasma glucose‡ (mmol/L)	0.0008	0.0006	0.156	0.2471	0.0014	0.0005	0.0058	0.5417
HbA1c‡ (%)	0.0189	0.0133	0.1544	0.1028	0.073	0.0146	6.47×10^{-7}¶	0.7476
Insulin resistance surrogate markers								
TyG§, ‡	8.52×10^{-7}	7.31×10^{-7}	0.2443	0.7013	3.00×10^{-6}	1.00×10^{-6}	0.0015	0.4966
TyG-BMI§, ‡ ($\times 10^3$)	2.54×10^{-8}	2.66×10^{-8}	0.3398	0.8221	1.16×10^{-7}	4.09×10^{-8}	0.0045	0.4196
TyG-WC§, ‡ ($\times 10^3$)	9.44×10^{-9}	7.72×10^{-9}	0.222	0.7286	4.05×10^{-8}	1.23×10^{-8}	0.001	0.3929
Atherosclerotic risk factors								
Diabetes mellitus (%)	1.962	0.6539	0.0027	0.7132	2.6383	0.8606	0.0022	0.7792
Hypertension (%)	2.9381	0.42	2.64×10^{-12}	0.016	3.2286	0.4906	4.67×10^{-11}	0.1135
Metabolic syndrome (%)	1.2252	0.4326	0.0046	0.9084	1.5966	0.4468	3.53×10^{-4}	0.8181

Abbreviations, adjusted condition and subjects recruited for analysis as in Figure 1 and Table 2

† hypertension, ‡ diabetes mellitus, and § hyperlipidemia.

*P***: adjusted for BMI, and current smoking and BMI: adjusted for smoking.

*P**: adjusted for age, BMI, and current smoking; Age: adjusted for BMI and current smoking; and BMI: adjusted for age and smoking

Significance between cg08097417 and studied phenotypes was defined as a *P* value of $< 0.05/(24+27) = 9.80 \times 10^{-4}$. *t*-test: ** $p < 0.001$, * $p < 0.01$.

Supplementary Table 6. Association of the cg08097417 methylation status with body-shape indices and metabolic traits according to obesity

Clinical and laboratory parameters	Non-obese (n = 1,018)				Obese (n = 618)			
	beta	SE	*P* value#	*P* value	beta	SE	*P* value#	*P* value
Age (years)	0.0151	0.0005	1.39×10^{-140}	-	0.0155	0.0006	7.36×10^{-99}	-
Body-shape indices								
Body height (cm)	-0.0096	0.0013	7.34×10^{-14}	0.5070	-0.0067	0.0015	1.00×10^{-5}	0.9647
Body weight (kg)	-0.0130	0.0017	1.34×10^{-13}	0.5870	-0.0070	0.0016	1.50×10^{-5}	0.8432
Hip circumference (cm)	-0.0113	0.0020	4.41×10^{-8}	0.2763	-0.0093	0.0023	6.10×10^{-5}	0.2145
Waist circumference (cm)	0.0051	0.0015	7.19×10^{-4}	0.6079	0.0091	0.0017	1.06×10^{-7}	0.1031
Waist hip ratio	0.9151	0.1348	1.92×10^{-11}	0.8669	1.4414	0.1710	2.50×10^{-16}	0.0150
Body fat percentage (%)	-0.0002	0.0030	0.9389	0.6812	-0.0008	0.0033	0.8165	0.7403
Body mass index (kg/m^2)	0.0133	0.0040	0.0010	0.5338	-0.0125	0.0036	5.45×10^{-4}**	0.2283
ABSI	0.0822	0.0150	5.72×10^{-8}	0.7272	0.1535	0.0207	4.17×10^{-13}*	0.0934
WHI	0.1956	0.0293	3.77×10^{-11}	0.8869	0.3321	0.0393	2.29×10^{-16}*	0.0136
HI	-0.0227	0.0157	0.1466	0.3989	-0.0310	0.0200	0.1213	0.1153
Blood Pressure								
Mean heart rate† (/min)	-0.0006	0.0009	0.5217	0.7655	-0.0017	0.0011	0.1295	0.7169
Systolic BP† (mmHg)	0.0045	0.0005	3.28×10^{-16}	0.0244	0.0036	0.0006	4.30×10^{-9}	0.3210
Diastolic BP† (mmHg)	0.0031	0.0009	2.82×10^{-4}	0.2730	0.0035	0.0009	1.67×10^{-4}	0.1010
Mean BP† (mmHg)	0.0047	0.0008	1.62×10^{-9}	0.0868	0.0043	0.0008	5.40×10^{-7}	0.1398
Lipid profiles								
Total cholesterol§ (mmol/L)	0.4552	0.0975	3.00×10^{-6}	0.9026	0.3092	0.1207	0.0107	0.0257
HDL cholesterol§ (mmol/L)	0.1224	0.0861	0.1554	0.9235	0.0822	0.1017	0.4195	0.7305
LDL cholesterol§ (mmol/L)	0.1944	0.0650	0.0029	0.8831	0.0908	0.0781	0.2455	0.0609
Triglyceride§ (mmol/L)	0.1360	0.0371	2.61×10^{-4}	0.8972	0.0772	0.0403	0.0563	0.4835
Glucose metabolism								
Fasting plasma glucose‡ (mmol/L)	0.0013	0.0006	0.0255	0.4838	0.0009	0.0005	0.0625	0.8094
HbA1c‡ (%)	0.0328	0.0136	0.0161	0.0459	0.0509	0.0139	2.65×10^{-4}	0.4458
Insulin resistance surrogate markers								
TyG§, ‡	3.00×10^{-6}	1.00×10^{-6}	0.0095	0.7942	1.00×10^{-6}	7.12×10^{-7}	0.1377	0.4039
TyG-BMI§, ‡ (× 10^3)	1.09×10^{-7}	4.41×10^{-8}	0.0135	0.7642	3.58×10^{-8}	2.49×10^{-8}	0.1516	0.4281
TyG-WC§, ‡ (× 10^3)	3.66×10^{-8}	1.29×10^{-8}	0.0046	0.7411	1.21×10^{-8}	7.32×10^{-9}	0.1004	0.3715
Atherosclerotic risk factors								
Diabetes mellitus (%)	2.0546	0.7376	0.0053	0.5157	2.3726	0.7453	0.0015	0.5319
Hypertension (%)	2.9773	0.4576	7.69×10^{-11}	0.0250	3.0894	0.4513	7.63×10^{-12}	0.1102
Metabolic syndrome (%)	1.7148	0.5420	0.0016	0.9195	1.0864	0.3829	0.0045	0.8752

Abbreviations, adjusted condition and subjects recruited for analysis as in Figure 1 and Table 2.*P*: adjusted for age, sex, BMI and current smoking and age: adjusted for sex, BMI and current smoking. † hypertension, ‡ diabetes mellitus, and § hyperlipidemia.
P#: adjusted for sex, BMI, and current smoking; Age: adjusted for BMI and current smoking; and BMI: adjusted for age and smoking.
Significance between cg08097417 and studied phenotypes was defined as a *P* value of < 0.05/(24+27) =

9.80× 10^{-4}
t-test : ** P < 0.001, * P < 0.01

Supplementary Table 7. Association of *KLF14* rs4731702 genotypes and cg08097417 methylation status with metabolically healthy and unhealthy phenotypes in obese Taiwan Biobank participants

MHO *vs.* MUO		N	OR	95%CI	Beta	SE	*P* value
rs4731702 genotypes	Total	29,891	0.88	0.85 - 0.92	-0.12	0.02	1.92 × 10^{-11}
cg08097417 methylation status	Total	633	4.04	1.95 - 8.41	1.40	0.37	1.81 × 10^{-4}

MHO: metabolically healthy obese, MUO: metabolically unhealthy obese, N: number, OR odds ratio, 95% CI: 95% confidence interval, SE standard error.
P: adjusted for age, sex, BMI and current smoking

Supplementary Figure 1. Regional association studies for genetic variants at positions between 130.3 and 130.5 mega-base on chromosome *7q32.2* for the high-density lipoprotein cholesterol level.

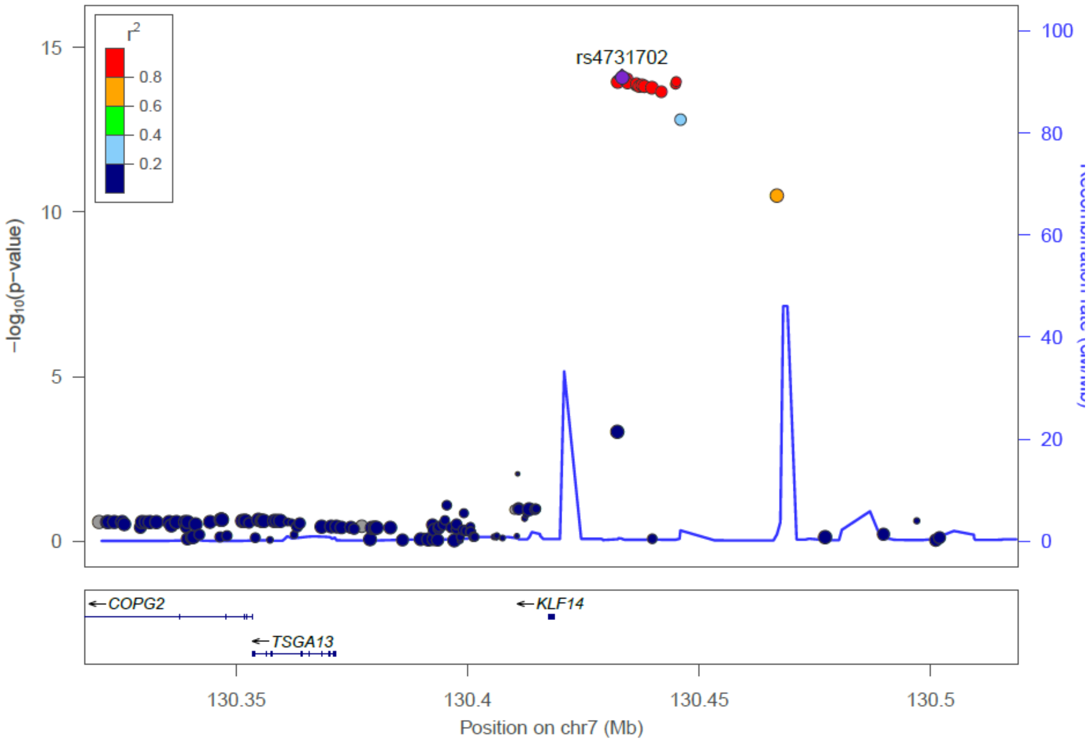

Supplementary Figure 2. Regional association studies for genetic variants at positions between 130.3 and 130.5 mega-base on chromosome *7q32.2* for triglyceride level.

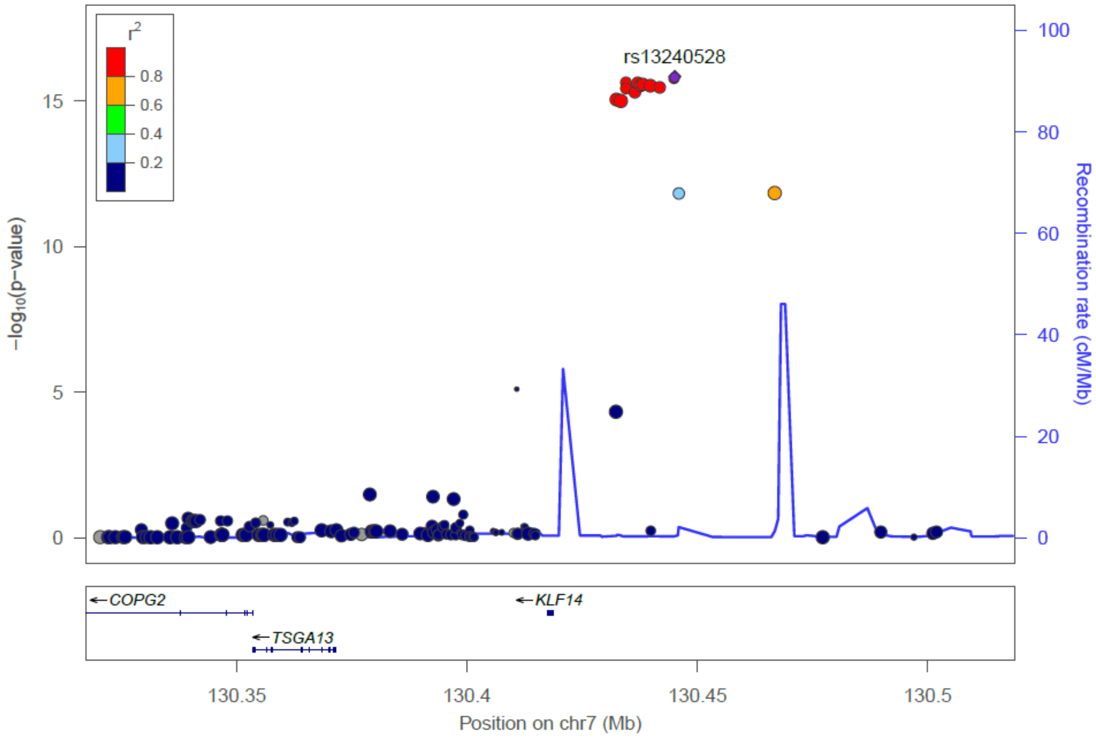

Supplementary Figure 3. Regional association studies for genetic variants at positions between 130.3 and 130.5 mega-base on chromosome *7q32.2* for the risk of metabolic syndrome.

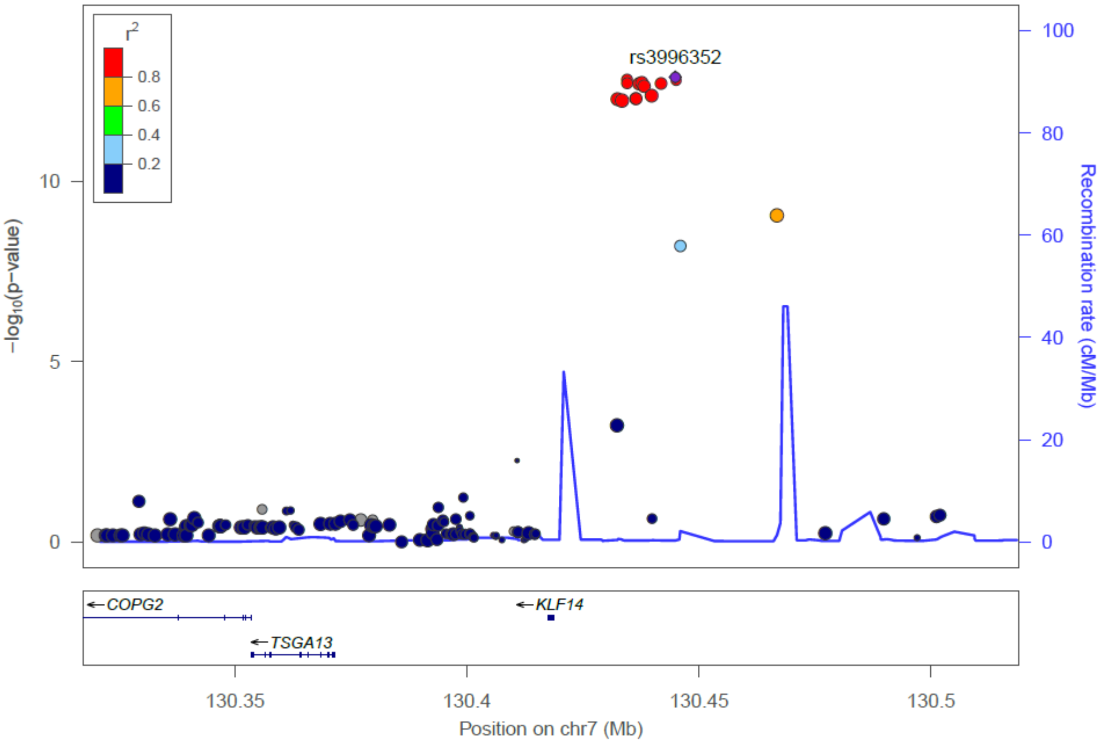

Supplementary Figure 4. Regional association studies for genetic variants at positions between 130.3 and 130.5 mega-base on chromosome *7q32.2* for a body-shape index.

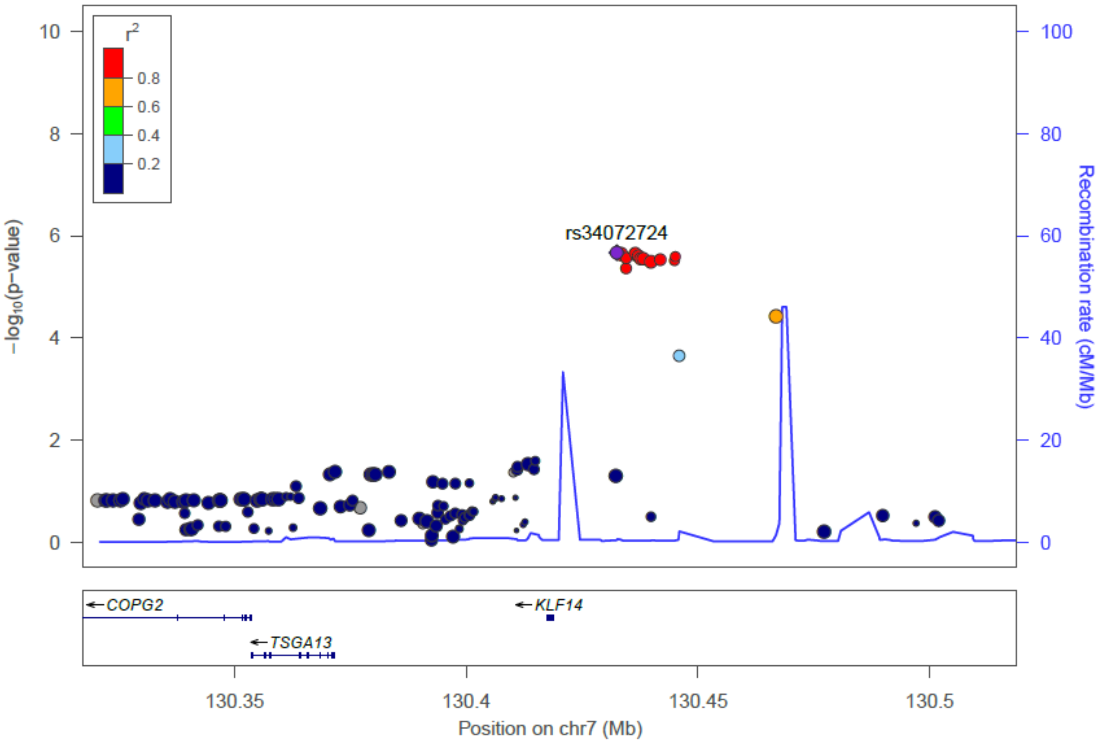

Supplementary Figure 5. Regional association studies for genetic variants at positions between 130.3 and 130.5 mega-base on chromosome *7q32.2* for body fat percentage.

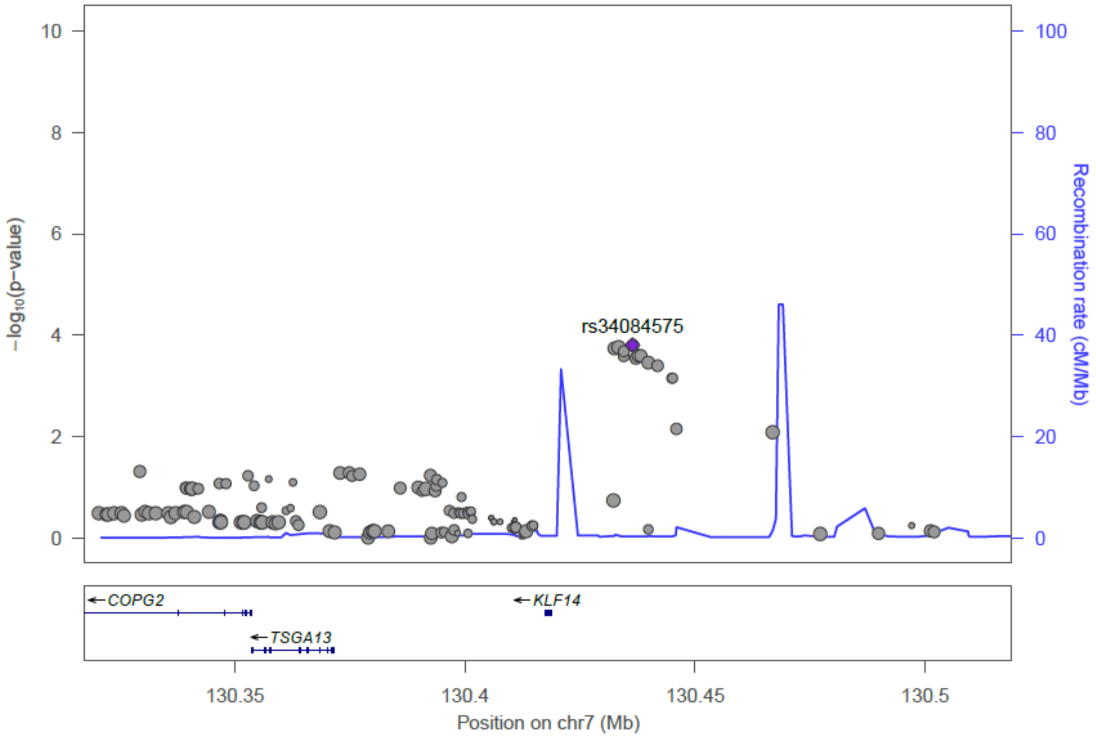

Supplementary Figure 6. Regional association studies for genetic variants at positions between 130.3 and 130.5 mega-base on chromosome *7q32.2* for body mass index.

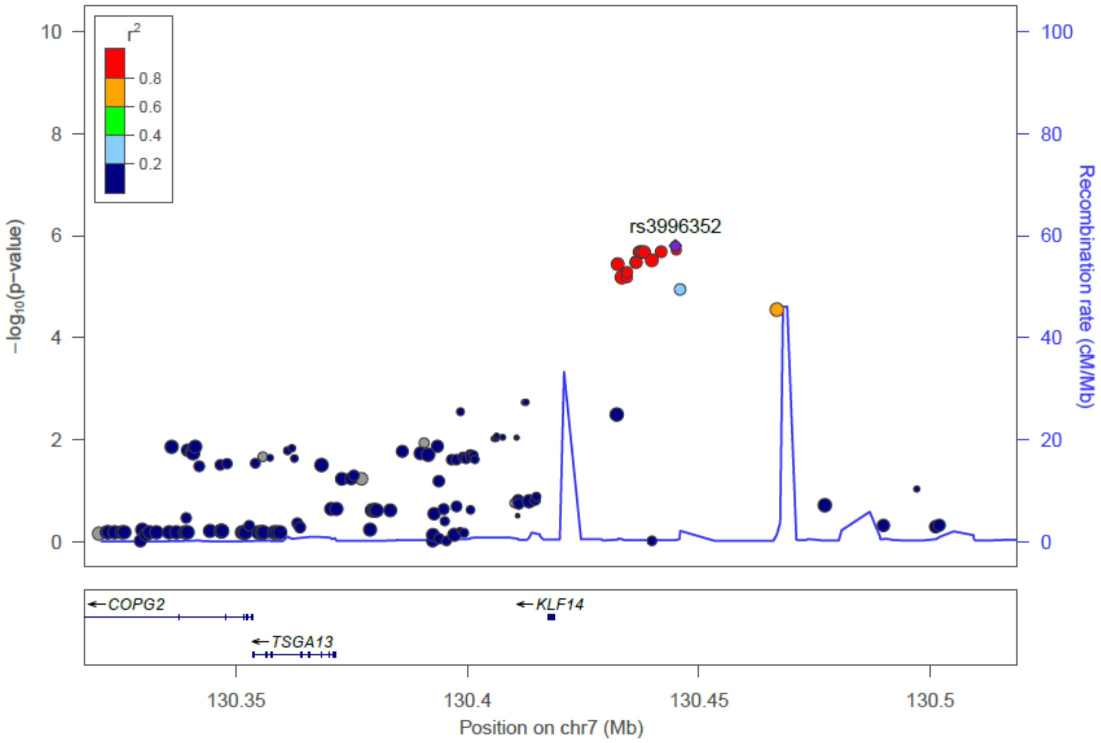

Supplementary Figure 7. Regional association studies for genetic variants at positions between 130.3 and 130.5 mega-base on chromosome *7q32.2* for hip circumference.

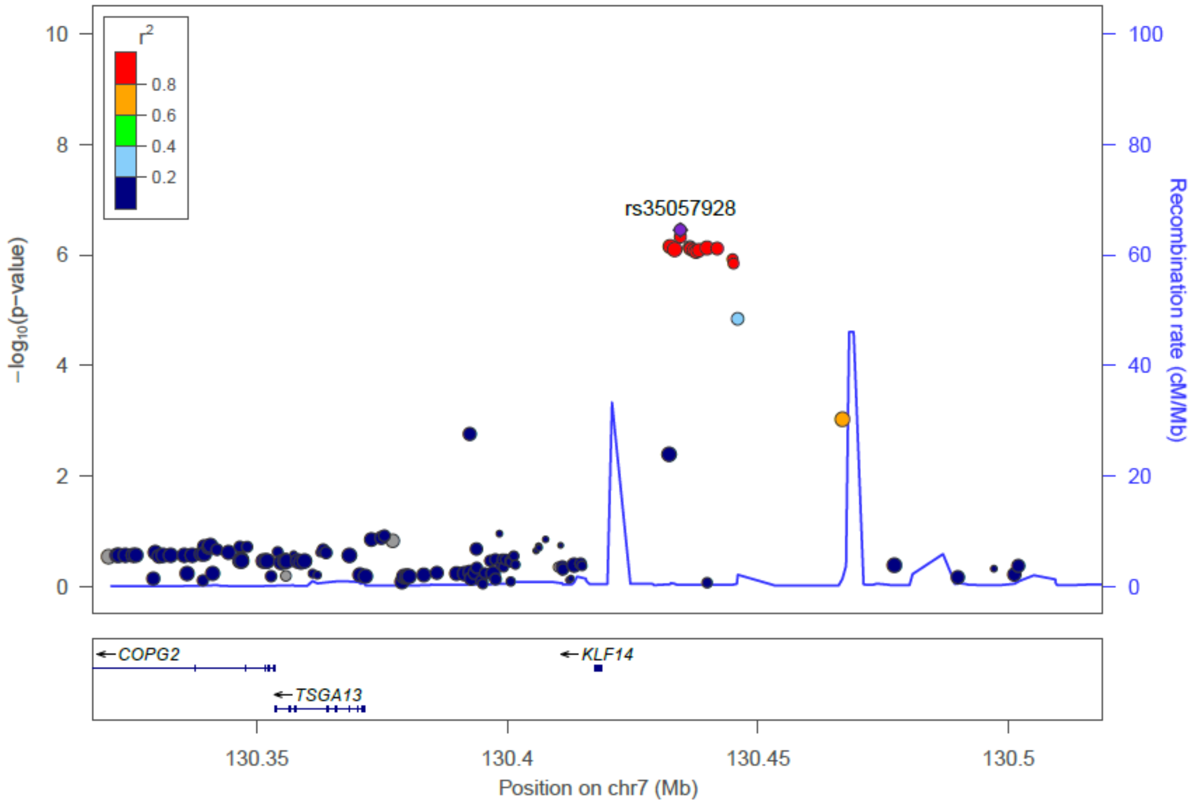

Supplementary Figure 8. Regional association studies for genetic variants at positions between 130.3 and 130.5 mega-base on chromosome *7q32.2* for hip index.

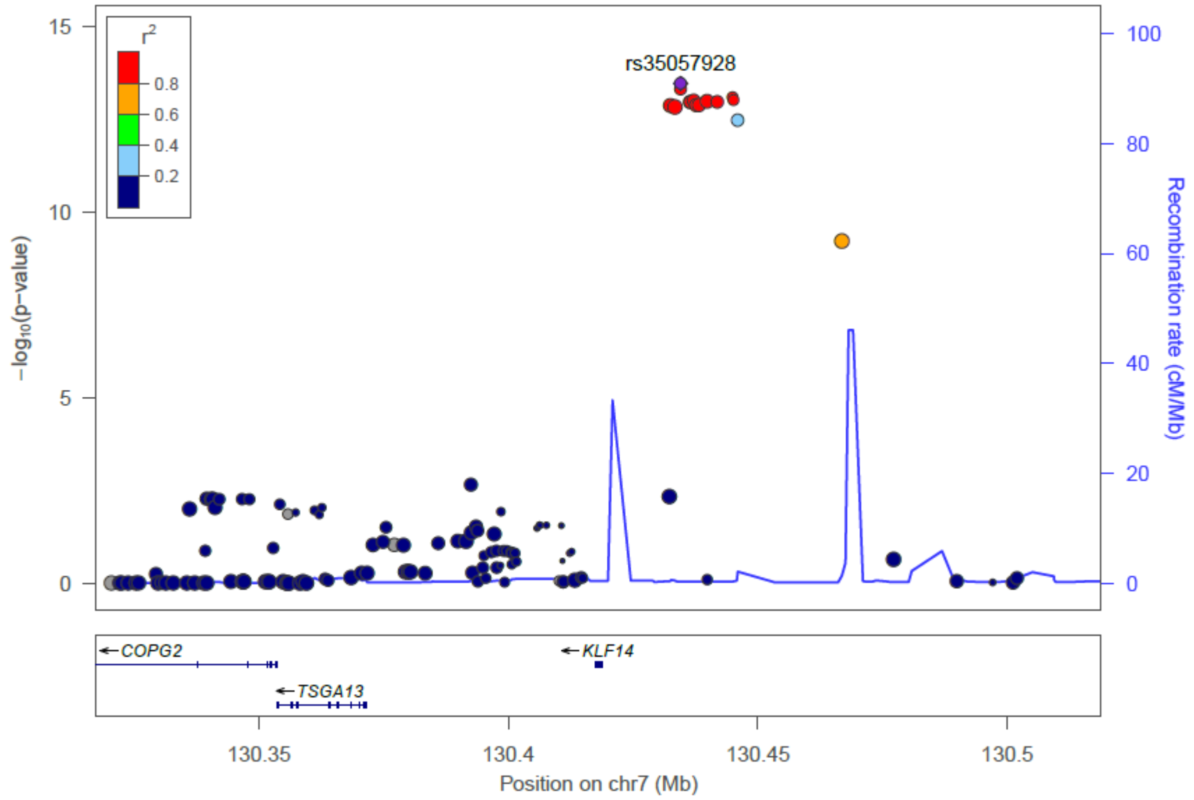

Supplementary Figure 9. Regional association studies for genetic variants at positions between 130.3 and 130.5 mega-base on chromosome *7q32.2* for mean blood pressure.

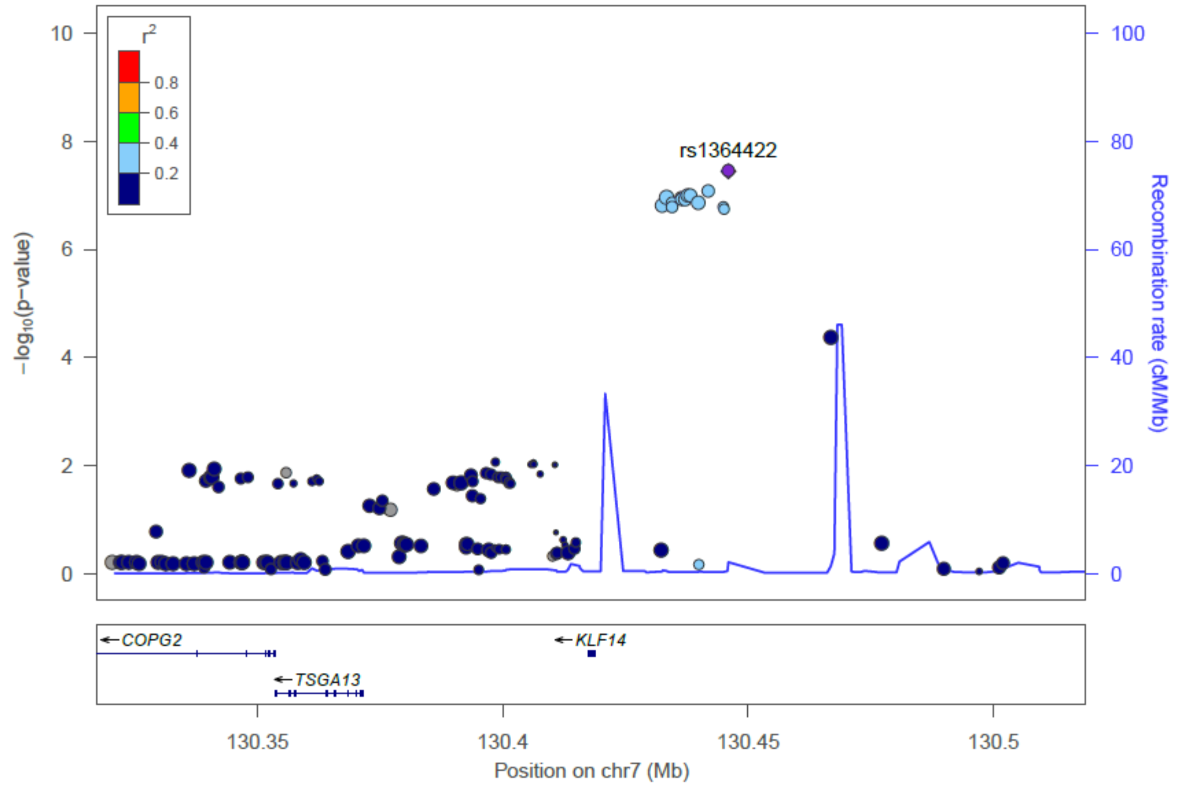

Supplementary Figure 10. Regional association studies for genetic variants at positions between 130.3 and 130.5 mega-base on chromosome *7q32.2* for waist circumference.

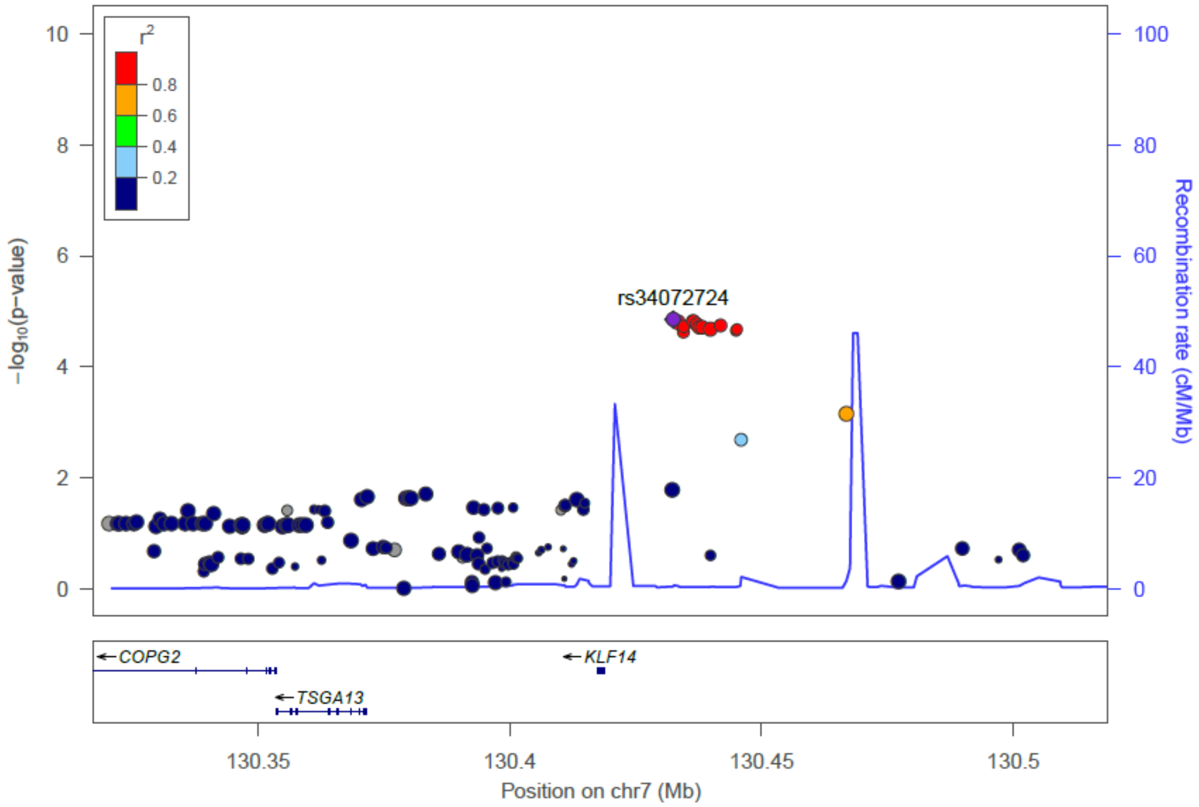

Supplementary Figure 11. Linkage disequilibrium map of the *KLF14* gene upstream region for lead single-nucleotide polymorphisms of the study phenotypes.

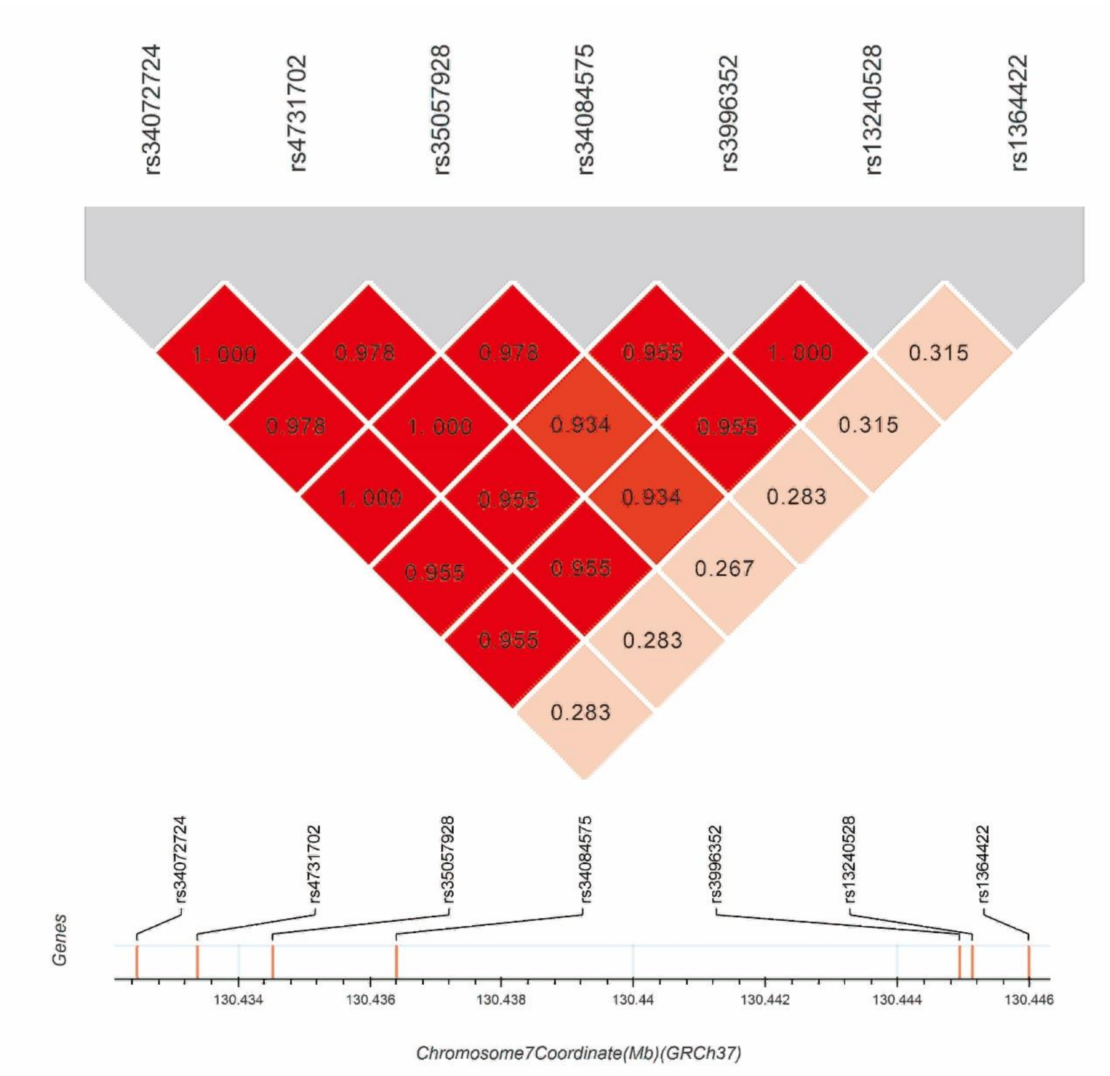

Supplement: Supplementary file 1 [file ijms-23-04165-s001.zip › ijms-1651976-supply.pdf]
